# Understanding digital health literacy in later life: the role of sociodemographic and health-related factors – a cross-sectional study

**DOI:** 10.1186/s12877-026-07485-9

**Published:** 2026-04-29

**Authors:** Franziska U. Jung, Melanie Luppa, Matthias Reusche, Kerstin Wirkner, Melanie Eberl, Yvonne Dietz, Christoph Engel, Steffi G. Riedel-Heller

**Affiliations:** 1 Institute of Social Medicine, Occupational Health and Public Health (ISAP), Leipzig University, Leipzig, Germany; 2 Institute for Medical Informatics, Statistics and Epidemiology, Leipzig University, Leipzig, Germany; 3 LIFE - Leipzig Research Centre for Civilization Diseases, Leipzig University, Leipzig, Germany

**Keywords:** Digital health literacy, Chronic disease, Body mass index, Health status

## Abstract

**Background:**

So far, digital health literacy (DHL) has been linked to disparities in access to digital tools, differences in usage habits, and varying levels of proficiency in using digital technologies. The aim of the current study was to investigate the link between sociodemographic or health-related factors and digital health literacy among older adults in Germany.

**Method:**

A total of 3,000 participants from the LIFE Adult Cohort – a population-based cohort study in Germany – aged 65 years and older were re-contacted and surveyed using a paper-and-pencil questionnaire in May 2024. Participation rate was 74%. A sample of *n* = 2.227 participants was analysed with regard to digital health literacy (revised eHealth Literacy Scale (GR-eHEALS)), sociodemographic and health-related factors (overall health status, chronic diseases and body mass index). In addition, participants were compared regarding their internet use, as well as application of health-related devices and services.

**Results:**

According to the multivariate regression analysis, digital health literacy (overall and sub-scales) was positively associated with better health status (*p* < 0.001). Having a chronic disease or long-term health problem, however, as well as BMI-category were not significantly related to DHL. Internet use was the most prominent predictor across all three models (*p* < 0.001). Being 75 years and older was significantly associated with a decrease in digital health literacy compared to being 65 to 69 years. Moreover, a high educational level (CASMIN) compared to a low level was associated with a 9.3% increase in digital health literacy. Whereas a high income (≥ 3.500 €) compared to an income below 1.500€ was associated with a 7.2% increase in digital health literacy. No significant associations were found with regard to sex or marital status.

**Conclusion:**

The findings of the current study highlight the importance of sociodemographic and health-related factors in relation to digital health literacy among individuals aged 65 years and older. Further research is needed to clarify these associations in terms of causality and underlying mechanisms in order to develop interventions that focus on older adults in order to improve digital health literacy.

**Supplementary Information:**

The online version contains supplementary material available at 10.1186/s12877-026-07485-9.

## Introduction

In the light of an aging society and an increase in the prevalence of chronic diseases worldwide, individual health behaviour is seen as an important determinant to promote and maintain good health. In this context, health literacy is highly relevant for the health and well-being of both individuals and society. People with higher health literacy are more capable of preventing illness, making health-promoting decisions, and actively managing their health [1]. They also navigate the healthcare system more effectively and communicate better with medical professionals [2]. As technology advances, digital health literacy is becoming increasingly important in the healthcare system, being acknowledged as “super social determinants of health” [3].

Digital health literacy (also referred to as “e-health literacy”) is defined as a dynamic, context-specific mix of individual and social skills as well as technological conditions. It may enable people to search for, evaluate, communicate, and apply digital health information in order to maintain or improve quality of life into old age [4–6]. The development of different definitions and concepts of digital health literacy and e-health literacy has been previously reviewed in greater detail [7]. Different types of digital information sources are distinguished, such as the internet, mobile health, or artificial intelligence. The ongoing digitalization of the healthcare system offers numerous opportunities for older adults (aged 65 and above), but also entails risks when digital health literacy is lacking. Many health-related pieces of information are now primarily available in digital formats, and the importance of digital sources has grown rapidly [4]. Given the rapid digitization in healthcare, enhancing digital health literacy is essential for older adults to navigate these changes, especially in managing chronic conditions and accessing healthcare resources effectively [8]. Without adequate digital health literacy, older adults may face barriers to accessing essential information and services. This not only limits their ability to benefit from digital health innovations but also risks widening technological divides, as those with lower skills become increasingly excluded from digital health systems.

Currently in Germany, about 56% of people aged 60 years and older access the internet using a smartphone, followed by notebooks (38.9%), computers (27.6%) and tablets (18.4%) [9]. The majority in this age group is accessing the internet several times a day (34.6%), once a day (10.8%) or several times during a week (13.8%), whereas 30.8% are non-users [10]. However, studies investigating determinants underlying the digital health divide find sources of inequalities that may specifically relevant to older adults: financial inequalities, societal level determinants and barriers to access and interaction with health technologies [11].

Systematic reviews indicate that older adults generally exhibit low levels of eHealth literacy — for example, an average score of 21.45 points on the eHealth Literacy Scale, where the maximum achievable score is 32 [12, 13]. Particularly affected groups include women, the oldest old (aged > 80 years), individuals living alone, and those from countries with low levels of digitalization. A generational divide in digital technology use has been observed previously, with older adults less likely to adopt digital health devices and technologies, particularly those with lower digital health literacy [14, 15]. Recent meta-analyses confirm that higher digital health literacy among older adults is positively associated with health-promoting behaviours, self-care, medication adherence, health knowledge, and improved decision-making [13]. Similarly, digital literacy has been recently linked to improved health outcomes among adults aged 65 years and older [16]. In this study, high IT use was significantly related to engagement in healthy lifestyle behaviour (e.g. decreased alcohol consumption and exercise). However, associations between digital or eHealth literacy and psychosocial outcomes have been shown to be inconsistent (positive, negative an not associations) [13], which may be explained by the limited measurement of digital health literacy in general [17].

Despite growing recognition of its importance, significant research gaps remain. Many existing studies primarily focus on access to digital technologies or usage frequency, rather than on the impact of digital health literacy on concrete health outcomes. Particularly underrepresented are the oldest old (those aged 80 and above), individuals living alone, and those with low socioeconomic status—groups that often face multiple barriers, such as digital exclusion and low levels of basic education. Research examining the relationship between digital health literacy and sociodemographic as well as health-related characteristics in older populations could help address these gaps. Such insights would support the identification of specific needs among sub-groups and the development of targeted interventions and strategies. These efforts could help bridge the “digital divide” and ensure equal access to health information and healthcare services for all individuals aged 65 and older. Therefore, the aim of this cross-sectional analysis was to investigate the association between digital health literacy and sociodemographic as well as health-related characteristics in German adults aged 65 years and older, using data from a population-based cohort survey.

## Method

### Data collection

The present study is a cross-sectional study. A total of *n* = 3,000 participants from the LIFE Adult Cohort were re-contacted and surveyed using a paper-and-pencil questionnaire. Information with regard to the recruitment and sampling procedure of the LIFE Adult cohort has been described in greater details elsewhere [18, 19]. The Life Adult cohort is a population-based cohort study, which included randomly selected participants (age range at study start: 40–79 years) from the city of Leipzig (Germany). The aim of this research project was to investigate prevalences, predispositions, and the relevance of lifestyle factors of major civilization disease, such as depression or metabolic disorders. Only those participants who were at least 65 years old at the time of the current survey (May 2024) were contacted. The response rate of the current study was 74.2% (*n* = 2.227). Informed consent was obtained from all participants beforehand. The study is conducted in accordance with the Declaration of Helsinki and received approval by the Ethics Committee of the Medical Faculty of Leipzig University (approval numbers 263–2009-14122009, 263/09-ff, 201/17-ek).

### Instruments

The following instruments and constructs were included.

With regard to sociodemographic characteristics, sex was surveyed using the answer categories male or female, whereas age was categorized into the following five age groups to allow for direct comparison: 65–69 years, 70–74 years, 75–79 years, 80–84 years, 85 years and older. Education was assessed by sociodemographic items on highest school qualification and highest job training qualification. Based on the answers, the following CASMIN^1^ categories were obtained: low (no or lower secondary education without vocational qualification), medium (intermediate or upper secondary education and/or vocational training), and high (tertiary education, including university or university of applied sciences degrees). The procedure has been described elsewhere [20]. Family status was categorized into three groups: single, married, divorced/widowed/separated. Household net income (monthly) was assessed using the following answer categories: <1500€, 1500 - <2000€, 2000 - <2500€, 2500 - <3000€, 3000 - <3500€, more than 3500€. *These categories were chosen in order to cover a wide range of net income and reflect the distribution of household net incomes among individuals aged 65 years and older in Germany* [21].

Subjective overall health status was survey using the EQ-VAS scale. Participants were asked to rate their current health status on a scale between "0" (worst health status) and "100" (best health status) [22]. Frailty has been defined by exceeding a cut-off of 72 [23]. In addition, participants were asked "Do you have at least one long-term health problem or chronic illness?" (yes/no answer format [5]), . This question has been included in numerous European health surveys (European Commission 2003; Eurostat 2011, GEDA Germany Study 2012). Body Mass Index (BMI) was calculated using current body weight and height provided by the participants (formula: kg/m^2)^, using the formula provided by the World Health Organization [24]. The participants were categorized into underweight/normal weight (BMI ≤ 24.9 kg/m^2^), overweight (BMI 25–29.9 kg/m^2^) and obesity (BMI ≥ 30 kg/m^2^).

In order to survey internet usage with regard to health and well-being, participants were asked the following question in order to assess frequency and familiarity: “How often they were using the internet?” (ranging from never (0) to several times a day (7)) and how familiar they are with using the internet (ranging from not very familiar (0) to very familiar (3)). The items have been shown to be useful for studies investigating internet usage among individuals aged 60 years and older [25].

In addition, participants were asked how often they use the internet to seek information on health-related issues or in order to check medical diagnoses (ranging from never (0) to always (4) for both items). Another purpose was to investigate how often participants in this age group are using digital health-related information services. In order to do so, they have been given eight possible services, such as e-appointment services or telemedicine, that have been provided by previous research project on digital health literacy [26, 27]. The items on internet usage and information seeking behaviour can be found in the Appendix.

Digital Health Literacy (DHL) was investigated using the 8-item revised German eHealth Literacy Scale (GR-eHEALS) [28], which consists of a 2-factor structure with two sub-scales (information seeking and information appraisal, possible range for each scale: 4–20), confirming construct validity. All items were assessed on 5-point Likert scales (1 = strongly disagree to 5 = strongly agree). The items can be found in the appendix. In the current study, Cronbachs‘s alpha was 0.97 for the overall scale, 0.97 for the sub-scale *information seeking* and 0.94 for the sub-scale *information appraisal*. Overall, a higher score indicates greater digital health literacy.

### Statistical analysis

The data described above was analysed descriptively in terms of digital health literacy and (health-related) internet use. In addition to describing the sociodemographic composition of the sample, mean (SD) digital health literacy scores were calculated for each sociodemographic subgroup to illustrate descriptive differences across groups. Descriptive figures were used to visually illustrate the distribution of digital health literacy across key sociodemographic groups (sex and age groups); these figures are intended for descriptive purposes only and do not represent planned inferential comparisons. Moreover, chi-square tests, t-tests, and Kruskal Wallis tests, were calculated if appropriate. In addition, multivariate regression models were applied to investigate the relationship between digital health literacy (overall and sub-scales, dependent variable, continuous) and sociodemographic characteristics as well as health-related variables (independent variables). The sociodemographic variables included sex (0 = male; 1 = female), age (categorical, coding: 1 = 65–69, 2 = 70–74, 3 = 75–79, 4 = 80–84, 5 = 85+), education/Casmin (1 = low, 2 = intermediate, 3 = high), family status (1 = single, 2 = married, 3 = divorced/widowed/separated), income (1 = < 1500€, 2 = 1500 - <2000€, 3 = 2000 - <2500€,4 = 2500 - <3000€, 5 = 3000 - <3500€,6 = more than 3500€). Health-related variables included BMI (0 = underweight/normal weight, 1 = overweight, 2 = obesity), general health status (continuous, possible range: 0-100) and chronic disease/ long-term health issue (0 = no, 1 = yes). In addition, the variable "internet use “was dichotomized (0 = no; 1 = yes) and also added to the regression model. Variables were tested for multicollinearity beforehand using the variance inflation factor with no strong indication of multicollinearity. As the dependent variable (digital health literacy) was not normally distributed, generalized linear models were applied using a gamma distribution with a log link and robust standard errors. This model specification was considered most appropriate based on graphical inspection and the results of the modified Park test [29, 30]. We report exponentiated coefficients, which can be interpreted as percentage changes in expected digital health literacy per one-unit increase in each independent variable., 95% confidence intervals and p-values. For all analyses, a p-value of 0.05 or lower was used to indicate statistically significance. Model goodness-of-fit was further evaluated using Akaike’s Information Criterion (AIC). The software program Stata/SE 16.0 [31] has been used for all analyses.

## Results

### Sociodemographic and health-related characteristics

Sociodemographic and health-related characteristics of the overall sample (*n* = 2.227) and with regard to digital health literacy are summarized in Table 1. The age of the participants ranged between 65 and 91 years (median: 75; mean: 75.7; standard deviation: 6.5) and more than a half of the sample was female (*n* = 1203; 54%).

**Fig. 1:**
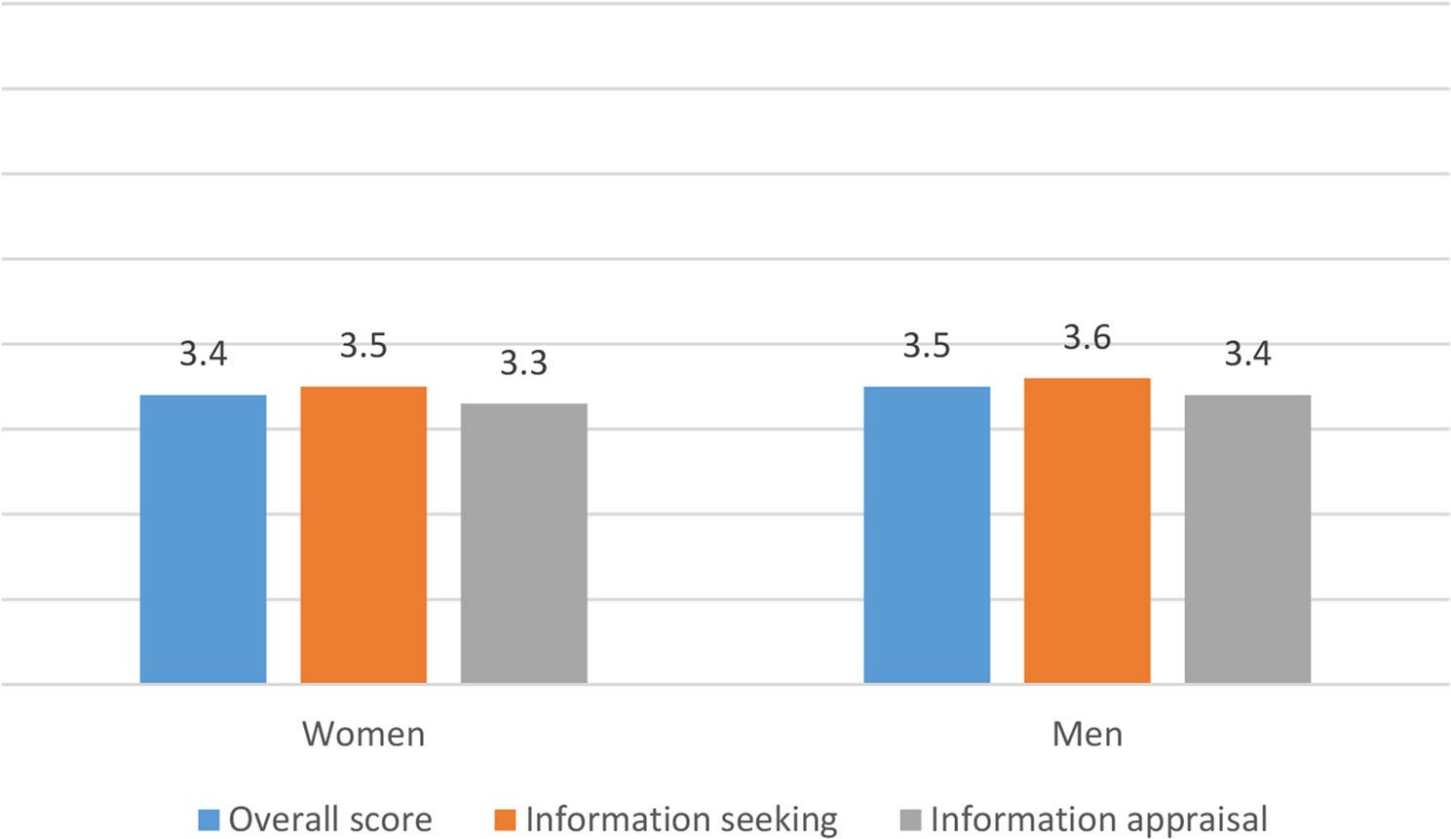
Digital health literacy (Overall and sub-scales) by sex (descriptive visualization) Note: Higher scores indicate greater digital health literacy (possible range: 1–5)

With regard to health, the mean Body Mass Index was approximately 27 kg/m^2^ (standard deviation: 5.0 kg/m^2^; median: 26.2 kg/m^2^; range: 13.4–71.6 kg/m^2^), 71.5% of the overall sample suffered from at least one chronic or long-term health condition and the mean score regarding overall health was 68.3 (standard deviation: 19.1; median: 71, range: 0-100).

### Digital health literacy

**Fig. 2:**
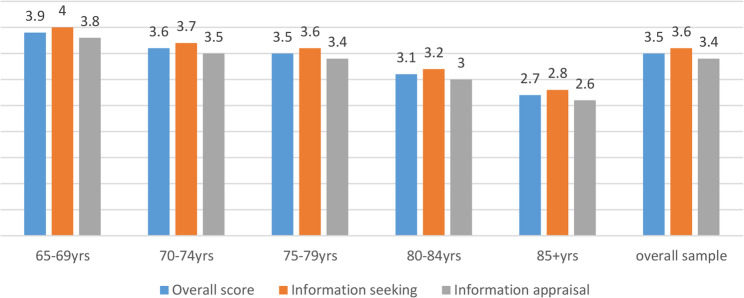
Digital Health Literacy (overall and sub-scales) by age (descriptive visualization) Higher scores indicate greater digital health literacy (possible range: 1–5)

The DHL score ranged from 1 to 5 (mean: 3.5; standard deviation: 1.3; median: 3.8) and significant differences were found with regard to age, educational attainment (CASMIN), family status, BMI-category, chronic health condition and overall health status/frailty (Table 1). In addition, the two sub-scales were also analysed. Information seeking ranged from 1 to 5 (mean: 3.6; standard deviation: 1.3; median: 4.0), similar to information appraisal (mean: 3.4; standard deviation: 1.3; median: 3.6). Both sub-scales did not differ in terms of sex, however, significant differences were found among age groups (*p* < 0.001 for both, Figs. 1 and 2). The item statistics of each item of the digital health literacy scale can be found in Appendix 1 (Tab. i.).

**Table 1:** Sociodemographic characteristics of the study sample and distribution of digital health literacy scores within sociodemographic subgroups (*n* = 2.227)

Variables		Total sample *n* (%)		Digital Health literacy M (SD)	*p*-Value (test statistics)^1^
Sociodemographic	Age				*p* < 0.001 (chi^2^ = 148.907)
	65–69		462 (20.8%)	7.76 (1.91)	
	70–74		574 (25.8%)	7.25 (2.38)	
	75–79		477 (21.4%)	6.97 (2.43)	
	80–84		491 (22.1%)	6.18 (2.67)	
	85 and older		223 (10.0%)	5.37 (2.70)	
	Sex				*p* = 0.873 (t = 0.881)
	female		1203 (54.0%)	6.87 (2.58)	
	male		1024 (46.0%)	6.96 (2.39)	
	Education (CASMIN)				*p* < 0.001 (chi^2^ = 159.996)
	low		268 (12.0%)	5.13 (2.67)	
	intermediate		983 (44.1%)	6.70 (2.53)	
	high		967 (43.4%)	7.55 (2.14)	
	Family status				*p* < 0.001 (chi^2^ = 17.862)
	single		95 (4.3%)	6.7 (2.7)	
	married		1407 (63.3%)	7.1 (2.4)	
	divorced/widowed/separated		722 (32.5%)	6.5 (2.7)	
	Income				*p* < 0.001 (chi^2^ = 96.096)
	< 1500 Euro		266 (11.9%)	5.9 (2.69)	
	1500–2000 Euro		316 (14.2%)	6.6 (2.59)	
	2000–2500 Euro		450 (20.2%)	6.7 (2.62)	
	2500–3000 Euro		406 (18.2%)	6.9 (2.37)	
	3000–3500 Euro		325 (14.6%)	7.2 (2.34)	
	> 3500 Euro		346 (15.5%)	7.9 (1.96)	
	missing		118 (5.3%)	5.9 (2.24)	
Health	BMI-Category				*p* = 0.002 (chi^2^ = 12.424
	underweight/normal weight		819 (36.8%)	7.1 (2.47)	
	overweight		893 (40.1%)	6.9 (2.47)	
	obesity		469 (21.1%)	6.7 (2.50)	
	missing		46 (2.1%)	6.3 (2.87)	
	Chronic disease or long-term health problem				*p* = 0.012 (t = 2.307)
	no		618 (28.6%)	7.1 (2.5)	
	yes		1547 (71.5%)	6.9 (2.5)	
	General Health Status				*p* < 0.001 (t = 11.346)
	No frailty		1137 (51.1%)	7.5 (2.3)	
	frailty		1090 (48.9%)	6.3 (2.6)	

^1^ using t-test or Kruskal-Wallis as appropriate; cases with missing information are not reported if they were below 1% of the overall sample, *M * Mean, *SD * Standard deviation

### Health-related internet use

In general, the majority (56%) is using the internet at least once a day (Table 2.). Overall, 61.5% of participants are feeling well familiar or very familiar while using the internet. In terms of health-related internet use, only a minority of participants search the internet always or mostly for information about illness (21.9%) or have been using the internet to check medical diagnoses (12.9%). Overall, there was a statistically meaningful association between digital health literacy and frequency of internet use, feeling familiar and health-related internet usage (Table 2). In other words, using the internet frequently, and feeling highly familiar was associated with greater digital health literacy. In addition, higher digital health literacy was more likely to be observed among participants, who reported searching the internet for information on illnesses or checking medical diagnoses more often compared to those who never or rarely do so.

**Table 2:** Internet use characteristics of the study sample and mean (SD) digital health literacy scores by internet use categories

Items regarding internet usage	*n* (%)	Digital Health literacy M (SD)	Significance
How often do you use the internet?^1^			
several times a day	995 (44.7%)	4.1 (0.8)	*p* < 0.001
once a day	251 (11.3%)	3.6 (1.0)	
several times a week	339 (15.2%)	3.6 (0.9)	
once a week	107 (4.8%)	3.0 (1.0)	
2–3 times a month	53 (2.4%)	3.1 (1.1)	
once a month	23 (1.0%)	3.0 (1.1)	
less than once a month	57 (2.6%)	2.3 (1.1)	
never	328 (14.7%)	1.5 (0.9)	
How familiar are you while using the internet?^2^			
not familiar	355 (15.9%)	1.6 (0.8)	*p* < 0.001
less familiar	435 (19.5%)	2.9 (1.0)	
well familiar	1057 (47.5%)	4.0 (0.8)	
very familiar	312 (14.0%)	4.4 (0.7)	
How often do you search the Internet for information about illnesses?^3^			
never	537 (24.1%)	2.0 (1.1)	*p* < 0.001
rarely	495 (22.2%)	3.5 (1.0)	
sometimes	638 (28.7%)	4.0 (0.8)	
mostly	370 (16.6%)	4.3 (0.7)	
always	117 (5.3%)	4.5 (0.7)	
How often do you use the Internet to check medical diagnoses?^4^			
never	922 (41.4%)	2.7 (1.3)	*p* < 0.001
rarely	538 (24.2%)	3.8 (0.8)	
sometimes	415 (18.6%)	4.1 (0.7)	
mostly	218 (9.8%)	4.3 (0.8)	
always	70 (3.1%)	4.5 (0.8)	

^1^ *n* = 74 missing cases (3.3%); ^2^ *n* = 68 missing cases (3.1%); ^3^ *n* = 70 missing cases (3.1%); ^4^ *n* = 64 missing cases (2.9%)

**Fig. 3:**
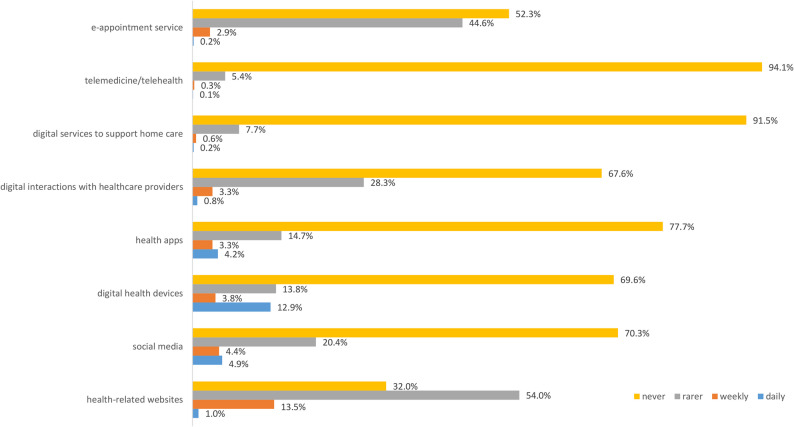
Frequency of use of digital health-related information services and devices (within a week)

In terms of health-related services or devices, results revealed that most participants rarely or never use them (Fig. 3). Overall, 21.1% (*n* = 470) did not use any of the health-related services or devices, whereas 42.1% (*n* = 938) have been using one service or device and 36.8% (*n* = 819) have been using two or more services and devices. Only health-related websites have been weekly (13.5%) or rarely (54.0%) used by the participants in this study, followed by e-appointment services (rarely: 44.6%; weekly: 2.9%), digital interactions with healthcare providers (rarely: 28.3%; weekly: 3.3%) or social media (rarely: 20.4%; weekly: 4.4%; daily: 4.9%). Digital health devices (i.e. wearable devices) have been used daily by 12.9% of participants (rarely: 13.8%; weekly: 3.8%).

Multivariate regression models revealed the following results with regard to digital health literacy, information seeking and information appraisal (Table 3). Across all three models, general health status was significantly and positively related to digital health literacy (Model 1 & 2: exp (b) = 1.002, *p* < 0.001, Model 3: exp (b) = 1.003, *p* < 0.001). In other words, a one-unit increase in general health status was associated with a 0.2% increase in digital health literacy and information seeking and a 0.3% increase in information appraisal. Having a chronic disease or long-term health problem as well as BMI category, however, were not significantly associated with better digital health literacy overall or with regard to information seeking or information appraisal. Internet use (yes/ no) was the most prominent predictor across all three models (*p* < 0.001, Table 3), showing that being an internet user (compared to a non-user) was associated with higher percentage increase in digital health literacy. With regard to sociodemographic factors, age and educational attainment (CASMIN) were significantly related to digital health literacy (overall, information seeking and appraisal). In other words, compared to being 65 to 69 years, being 75 years and older was associated with a reduction in digital health literacy. Moreover, a high educational level according to CASMIN compared to a low level was associated with a 9.3% increase in digital health literacy. Whereas a high income (≥ 3.500 €) compared to an income below 1.500€ was associated with a 7.2% increase in digital health literacy (Table 3). Being female or male, and marital status were not significantly associated with digital health literacy.

**Table 3:** Multivariate regression Models (GLM) with overall digital health literacy (Model 1), subscale "Information Seeking “(Model 2) and subscale "Information Appraisal “(Model 3) as the outcome variable

Variables	Model 1 Digital Health Literacy^1^		Model 2 Information Seeking^2^		Model 3 Information Appraisal^3^	
	Exp (b) (95% CI)	*p*	Exp (b) (95% CI)	*p*	Exp (b) (95% CI)	*p*
General Health Status (cont.)	**1.002 (1.001**,** 1.003)**	**< 0.001**	**1.002 (1.001**,** 1.003)**	**< 0.001**	**1.003 (1.002**,** 1.004)**	**< 0.001**
BMI-Category	chi^2^ (2) = 1.18, *p* = 0.553		chi^2^ (2) = 0.90, *p* = 0.637		chi^2^ (2) = 1.32, *p* = 0.518	
normal weight	*reference*		*reference*		*reference*	
overweight	0.983 (0.952, 1.015)	0.288	0.984 (0.953, 1.017)	0.348	0.981 (0.948, 1.015)	0.260
obesity	0.986 (0.946, 1.029)	0.523	0.989 (0.947, 1.033)	0.610	0.985 (0.942, 1.030)	0.509
Chronic disease or long-term health problem (ref.: no)	1.011 (0.977, 1.047)	0.530	1.022 (0.986, 1.059)	0.230	0.999 (0.963, 1.037)	0.959
Internet use (ref.: no)	**2.294 (2.130**,** 2.472)**	**< 0.001**	**2.383 (2.211**,** 2.570)**	**< 0.001**	**2.213 (2.049**,** 2.391)**	***p*** ** < 0.001**
Age	chi^2^ (4) = 13.79, *p* = 0.008		chi^2^ (4) = 15.05, *p* = 0.005		chi^2^ (4) = 11.14, *p* = 0.025	
65–69	*reference*		*reference*		*reference*	
70–74	0.976 (0.943, 1.009)	0.150	0.973 (0.940, 1.008)	0.129	0.979 (0.944, 1.015)	0.255
75–79	**0.954 (0.919**,** 0.990)**	**0.013**	**0.953 (0.917**,** 0.990)**	**0.014**	**0.956 (0.919**,** 0.995)**	**0.028**
80–85	**0.936 (0.893**,** 0.981)**	**0.006**	**0.930 (0.885**,** 0.977)**	**0.004**	**0.941 (0.896**,** 0.989)**	**0.015**
85+	**0.902 (0.839**,** 0.970)**	**0.005**	**0.896 (0.833**,** 0.962)**	**0.003**	**0.903 (0.835**,** 0.976)**	**0.010**
Sex (ref.: men)	1.002 (0.973, 1.032)	0.902	1.004 (0.973, 1.036)	0.792	0.999 (0.968, 1.031)	0.966
CASMIN	chi^2^ (2) = 12.96, *p* = 0.002		chi^2^ (2) = 12.09, *p* = 0.002		chi^2^ (2) = 11.63, *p* = 0.003	
low	*reference*		*reference*		*reference*	
intermediate	1.047 (0.975, 1.125)	0.207	1.054 (0.978, 1.136)	0.166	1.027 (0.954, 1.105)	0.478
high	**1.093 (1.020**,** 1.171)**	**0.011**	**1.098 (1.022**,** 1.179)**	**0.010**	**1.079 (1.005**,** 1.158)**	**0.036**
Marital status	chi^2^ (2) = 0.07, *p* = 0.968		chi^2^ (2) = 0.17, *p* = 0.917		chi^2^ (2) = 0.81, *p* = 0.666	
single	*reference*		*reference*		*reference*	
married	0.992 (0.917, 1.074)	0.846	1.013 (0.934, 1.100)	0.763	0.966 (0.893, 1.047)	0.400
divorced/widowed/separated	0.990 (0.914, 1.072)	0.802	1.005 (0.926, 1.092)	0.900	0.977 (0.9012, 1.058)	0.570
Income	chi^2^ (5) = 11.27, *p* = 0.046		chi^2^ (5) = 12.05, *p* = 0.034		chi^2^ (5) = 8.97, *p* = 0.110	
< 1500€	*reference*		*reference*		*reference*	
1500 - <2000€	1.062 (0.991, 1.138)	0.087	1.074 (1.000, 1.154)	0.049	1.047 (0.976, 1.124)	0.196
2000 - <2500€	1.034 (0.975, 1.097)	0.264	1.038 (0.976, 1.104)	0.236	1.034 (0.972, 1.100)	0.290
2500 - <3000€	1.014 (0.954, 1.078)	0.649	1.019 (0.957, 1.086)	0.553	1.013 (0.951, 1.079)	0.687
3000 - <3500€	1.052 (0.986, 1.123)	0.125	1.053 (0.984, 1.126)	0.136	1.057 (0.987, 1.132)	0.110
≥ 3500€	**1.072 (1.008**,** 1.140)**	**0.026**	**1.078 (1.012**,** 1.149)**	**0.020**	**1.071 (1.005**,** 1.142)**	**0.036**
	R^2^ = 0.434 AIC = 5.822		R^2^ = 0.423 AIC = 4.482		R^2^ = 0.391 AIC = 4.380	

*cont*. continuous variable, *exp (b)* exponentiated coefficients, can be interpreted as percentage changes in expected DHL per one-unit increase of the variable, *BMI * Body Mass Index

bold indicates significance (*p* < 0.05)

^1^*n* = 1.831

^2^*n* = 1.848

^3^*n* = 1.869

## Discussion

In recent years, digital health literacy has emerged as a critical component of healthcare access and management, particularly as digital tools and platforms are becoming increasingly integrated into health systems. However, disparities persist in the levels of digital literacy among older adults, influenced by various factors. Currently, there is a lack of research, addressing underlying social and health-related determinants affecting access to digital information services. While previous studies have examined DHL, evidence regarding its association among older adults remains limited and inconsistent. The present study addresses this gap by providing a comprehensive analysis of age and DHL.

According to the current results, DHL score ranged from 1 to 5 (median: 3.8), suggesting a medium level of DHL, comparable to other studies [32–34]. The mean values of the two sub-scale are also comparable to another recent study, which reported a mean score of 3.7 for information seeking (current study: 3.6) and a mean score of 3.6 for information appraisal (current study: 3.4) among individuals aged 65 years and older [32]. Multivariate regression analysis revealed that internet use (yes or no) was the most prominent predictor of DHL. Moreover, better health status, higher household income and higher educational attainment were related to an increase in digital health literacy, whereas having a chronic disease or long-term problem was not associated with DHL. With regard to sociodemographic characteristics, being 75 years and older was related to a decrease in digital health literacy. Family status, sex or BMI-category however, were not significantly related to digital health literacy within regression analysis. Previous studies have also demonstrated that lower levels of digital health literacy may be associated with being older and lower levels of education, as well as lower income [8, 35, 36]. Age-related effects may stem from factors such as cognitive decline and sensory impairments, as well as differences in economic development and cultural contexts, suggesting that these effects are not necessarily due to age itself [7, 36]. Furthermore, it has been suggested that higher income may lead to older adults having a higher number of electronic devices [37]. In the context of gender differences, one study showed that women report higher competencies with regard to information seeking, but not information appraisal [38], whereas the majority of studies did not find any gender differences [39], similar to our findings.

Studies investigating the relationship between health status and digital health literacy are scarce and previous findings are rather mixed. According to the current results, chronic disease and long-term problems are not related to digital health literacy, which has been found before [38]. Older adults with chronic diseases often rely on established care pathways and long-standing relationships with health care providers, reducing the need to search for digital health information independently. On the other hand, physical, cognitive, or sensory restrictions associated with chronic conditions or health issues may hinder the use of digital technologies, counterbalancing any increased motivation to seek (digital) health information. From a public policy perspective, these findings suggest that interventions should go beyond access and motivation. To prevent widening digital divides, strategies should emphasize training in evaluation skills and support for safe navigation of digital health environments, particularly for older adults and those with chronic conditions. One Example could be to integrate digital literacy into chronic disease management programs and offer low-cost access and devices to older adults. Future studies could investigate potential mediating or moderating factors to clarify the relationship between chronic diseases and digital health literacy.

In the present study, self-reported overall health status was positively related to digital health literacy. The association between better health status and higher digital health literacy may underline the effects of digital divide, where individuals with more resources and stable health are better positioned to access and benefit from digital health information [40]. In contrast, individuals with fewer resources may face compounded disadvantages, including lower access to devices and internet, less familiarity with digital environments, and reduced cognitive capacity due to health burden.

In addition, several additional factors may contribute to variations in DHL among older adults. These include prior exposure to digital technologies in the workplace, individual interest in digital innovations, and perceived usefulness of digital health applications. In countries with well-functioning health systems, such as Germany, previous findings indicate that despite an expressed interest in digital technologies, older adults often prefer analogue forms of consultation over video-based care [14, 41]. Future research should disentangle how individual, occupational, and system-level factors interact to influence DHL in later life.

The current study also exploratory investigated health-related use of digital devices and internet use. In summary, internet use was reported on a daily basis by 56% of the participants in this study, which is slightly higher (51%) compared to a survey conducted in Germany [10]. In this context, 62% felt well or very familiar with using the internet. In terms of health-related internet use, only a minority of participants search the internet al.ways or mostly for information about illness (21.9%) or have been using the internet to check medical diagnoses (12.9%). Overall, internet use, feeling familiar and health-related internet use was positively related to digital health literacy. In other words, digital health literacy was greater in individuals, who use the internet more often in in general and in order to search for health-related information on diseases or check medical diagnoses. This is also reflected in the frequency of use of certain health-related digital services and devices, which is significantly lower compared to other studies with younger participants. In this context, study participants only occasionally used e-appointment services and health-related websites. In a study by Schaeffer et al. [26], health-related websites were used more often compared to other services or tools. In comparison, another study revealed greater use of health-related apps as well as websites [34]. Regarding attitudes and interest in using telemedicine, the three most influential factors linked to the willingness to use it were confidence in its security, the perceived convenience it offers, and sufficient eHealth literacy [35]. These factors may be generalized to the usage of other digital health devices; however, more research is needed to investigate barriers of individual health-related digital services and devices.

Due to the cross-sectional character of the study, it is not possible to draw conclusion about the direction or causality of the association between digital health literacy and the use of digital devices or health-related information platforms. Previous studies have shown that the number of digital devices that have been used, as well as general greater use of digital health-related information platforms were independently associated with digital health literacy scores [42]. Although the study by Zhao et al. included only 2.1% of individuals aged 65 and older, this finding may not be a question of age. Individuals with higher levels of overall digital health literacy may have easier access to digital information and experience fewer fears, or may find their way around more quickly and easily. Frequent use of digital information services, on the other hand, may lead to improved health literacy, because it may lead to more “training possibilities”. Contrary, barriers related to low digital health literacy may hinder effective usage of these facilities or devices. Future studies employing longitudinal designs are needed to disentangle these reciprocal relationships and to clarify causal pathways.

The question remains, how health status may influence digital health literacy. In general, one would expect that individuals who suffer from chronic diseases or health issues have a greater motivation to look for health-related information. On the other hand, health issues may be caused by lack of (digital) health literacy, if individuals are not well informed about prevention and healthy lifestyle possibilities. Conversely, poor physical health—particularly impairments in cognitive functioning, vision, and hearing—should not be regarded as a barrier to the use of digital health technologies. Instead, these technologies must be adapted to accommodate the specific needs of older adults, particularly in terms of how information is presented [11]. In addition, a lack of trust (i.e. misuse of privacy data by third parties) represents a significant barrier to the adoption and effective use of digital health tools, particularly among older adults [43].

### Strengths and limitation

The study has drawbacks, which should be kept in mind, when interpreting the results – especially in a broader context and in comparison, to other samples. Overall, the sample includes participants from rural as well as urban areas in the eastern part of Germany, however, results may not be representable to other regions within Germany or abroad. Additionally, earlier analyses of the LIFE-Adult-Study suggest that baseline participation was more common among individuals with higher socioeconomic standing, healthier behavioural patterns, and a reduced prevalence of disease [44]. Prospective studies may want to investigate the link between digital health literacy and sociodemographic or health-related factors using a more diverse sample. In addition, a subjective measure has been used in order to investigate or assess health-related information (i.e. overall health status, weight and height). In this context, it has been shown that older women and men tend to overestimate their weight and height, leading to misclassification of BMI [45]. Despite its widespread use, the assessment of digital health literacy remains conceptually and methodologically challenging [17]. Digital health literacy encompasses a broad range of competencies, including not only information seeking and appraisal but also skills related to content creation, communication, contextual understanding, and critical reflection within digital environments. While the revised German eHealth Literacy Scale (GR-eHEALS) provides a validated and pragmatic measure, it primarily captures perceived abilities related to accessing and evaluating online health information. As such, it may not fully reflect the multidimensional nature of digital health literacy as described in more recent theoretical frameworks. In addition, participants may overestimate or underestimate their actual abilities or may have difficulties assessing their own skills with regard to health literacy [46]. Consequently, the findings related to digital (health) literacy in this study should be interpreted with caution, acknowledging the measurement constraints of the instrument. Future research would benefit from the development and validation of more comprehensive tools that better align with the evolving conceptualization of digital health literacy. A key strength of this study is the large and heterogeneous sample of older adults, enhancing the generalizability of the findings within this age group. By including community-dwelling older adults rather than clinical samples only, this study provides insight into digital health literacy under everyday conditions. Additional strengths include the use of a validated digital health literacy measure and the adoption of a multidimensional analytical approach. By focusing on an underrepresented population and examining digital health literacy within the context of the German health care system, the study provides findings that are both methodologically robust and practically relevant.

### Implications

A significant body of literature reported strategies and interventions in order to improve digital health literacy among older adults [35]. Specific training programs tailored for older adults and supported by community resources, have shown promise in enhancing their digital competencies. Programs designed with input from seniors can lead to improved e-health literacy by promoting an understanding of the technology and the context in which it is applied (Kim et al., 2023; Omar et al., 2023). For example, initiatives that encourage collaborative learning, as highlighted by Xie, demonstrate that peer engagement can be a powerful catalyst for enhancing digital health literacy among older adults (Xie, 2011). Recent reviews support these findings and identify effective strategies to enhance eHealth literacy among older adults, including theory-based training programs (e.g., IMB models), mentoring programs (intergenerational or peer coaching), online courses, and practical training sessions [12, 13, 47]. In Germany, the “DigitalPakt Alter” promotes low-threshold learning opportunities and hands-on training, for example through target group–oriented institutions. So far, digital health literacy has rarely been systematically addressed in routine care (e.g., by general practitioners, health insurance providers, or adult education centres). Besides, there is a lack of intersectoral strategies to embed digital health education in nursing homes, senior centres, or adult education programs. Clearly, more research is needed in order to develop effective tools and training programs that aim to improve digital health literacy – especially among older adults.

## Conclusion

The current study underlines the relevance of certain sociodemographic factors (age, education and income) as well as health-related aspects (general health status and chronic disease/long-term health problem) in the context of digital health literacy. In other words, digital health literacy decreased significantly with age. Moreover, higher educational attainment and higher household income was related to greater digital health literacy. With regard to health, a better overall health status was related to greater digital health literacy. More research is needed to investigate the relationship between chronic disease and digital health literacy. Future studies could focus on other determinants, such as affinity for technology, social support or the relevance of resilience or self-efficacy, in order to draw a complete picture of digital health literacy among silver agers.

## Supplementary Information

Supplementary Material 1.

## Data Availability

Due to privacy and ethical restrictions the data is available upon reasonable request from the corresponding author.

## Footnotes

1 Comparative Analysis of Social Mobility in Industrial Nations.
