# Correction: Understanding digital health literacy in later life: the role of sociodemographic and health-related factors – a cross-sectional study

**DOI:** 10.1186/s12877-026-07759-2

**Published:** 2026-06-09

**Authors:** Franziska U. Jung, Melanie Luppa, Matthias Reusche, Kerstin Wirkner, Melanie Eberl, Yvonne Dietz, Christoph Engel, Steffi G. Riedel-Heller

**Affiliations:** 1 Institute of Social Medicine, Occupational Health and Public Health (ISAP), Leipzig University, Leipzig, Germany; 2 Institute for Medical Informatics, Statistics and Epidemiology, Leipzig University, Leipzig, Germany; 3 LIFE - Leipzig Research Centre for Civilization Diseases, Leipzig University, Leipzig, Germany

**Correction: BMC Geriatr 26, 606 (2026)**

10.1186/s12877-026-07485-9

Following publication of the original article [1], the authors reported that the citation of Table 1 was incorrectly cited in the text as Fig. 1. The corrected text is highlighted in bold below.

**Incorrect**

Sociodemographic and health-related characteristics of the overall sample (n = 2.227) and with regard to digital health literacy are summarized in **Fig. 1**. The age of the participants ranged between 65 and 91 years (median: 75; mean: 75.7; standard deviation: 6.5) and more than a half of the sample was female (n = 1203; 54%).

**Correct**:

Sociodemographic and health-related characteristics of the overall sample (*n* = 2.227) and with regard to digital health literacy are summarized in **Table 1**. The age of the participants ranged between 65 and 91 years (median: 75; mean: 75.7; standard deviation: 6.5) and more than a half of the sample was female (*n* = 1203; 54%).

The original article has been corrected.
