# Supplementary material for: Understanding digital health literacy in later life: the role of sociodemographic and health-related factors – a cross-sectional study
Source: BMC Geriatr. 2026 Apr 29;26:606. doi: 10.1186/s12877-026-07485-9 (PMC13130565; doi:10.1186/s12877-026-07485-9)
Supplement: Supplementary file 1 — Supplementary Material 1. [file 12877_2026_7485_MOESM1_ESM.docx]

Appendix 1

Item statistics of the digital health literacy scale (GR-eHEALS)

| **Item** | **mean** | **standard deviation** |
| --- | --- | --- |
| I know how to find helpful health resources on the Internet.^1^  (German:  *Ich weiß, wie ich Internetseiten mit hilfreichen Gesundheitsinformationen finden kann.*) | 3.63 | 1.40 |
| I know how to use the Internet to answer my health questions.^1^  (German: *Ich weiß, wie ich das Internet nutzen kann, um Antworten auf meine Gesundheitsfragen zu erhalten*.) | 3.63 | 1.40 |
| I know what health resources are available on the Internet.^1^  (German: *Ich weiß, welche Seiten mit Gesundheitsinformationen im Internet verfügbar sind*.) | 3.41 | 1.38 |
| I know where to find helpful health resources on the Internet.^1^  (German: *Ich weiß, wo ich im Internet hilfreiche Gesundheitsinformationen finden kann*.) | 3.49 | 1.36 |
| I know how to use the health information I find on the Internet to help me.^2^  (German: *Ich weiß Gesundheitsinformationen aus dem Internet so zu nutzen, dass sie mir weiterhelfen.)* | 3.30 | 1.34 |
| I have the skills I need to evaluate the health resources I find on the Internet.^2^  (German: *Ich bin in der Lage, Internetseiten mit Gesundheitsinformationen kritisch zu bewerten*.) | 3.54 | 1.36 |
| I can tell high-quality from lowquality health resources on the Internet.^2^  (German: *Ich kann zwischen vertrauenswürdigen und fragwürdigen Internetseiten mit Gesundheitsinformationen unterscheiden*.) | 3.37 | 1.36 |
| I feel confident in using information from the Internet to make health decisions.^2^  (German: *Ich fühle mich sicher darin, Informationen aus dem Internet zu nutzen, um Entscheidungen in Bezug auf meine Gesundheit zu treffen*.) | 3.22 | 1.39 |

Note: ^1^ items of the subscale information seeking; ^2^ items of the subscale information appraisal

Items with regard to internet usage:

1. How often do you use the internet? (German: *Wie häufig nutzen Sie das Internet*?“)

0 = never

1 = less than once a month

2 = once a month

3 = 2-3 times a month

4 = once a week

5 = several times a week

6 = once a day

7 = many times a day

2. How familiar do you feel when using the internet? (German: *Wie vertraut fühlen Sie sich selbst im Umgang mit dem Internet?*)

0 = not very familiar

1 = little familiar

2 = well familiar

3 = very familiar

3. Assume you have health problems. Please indicate how often you search for information about illnesses on the internet? (German: *Angenommen Sie haben gesundheitliche Beschwerden. Bitte geben Sie an, wie häufig Sie Informationen zu Krankheiten über das Internet suchen.*“)

0 = never

1 = rarely

2 = sometimes

3 = mostly

4 = always

4. If you are under medical treatment: How often do you use the internet to check your medical diagnosis? (German: *Wenn Sie in ärztlicher Behandlung sind: Wie häufig nutzen Sie das Internet, um die ärztliche Diagnose zu prüfen?)*

0 = never

1 = rarely

2 = sometimes

3 = mostly

4 = always

5. On how many days of a typical week do you use the following digital health-related information services? (German: An wie vielen Tagen einer typischen Woche nutzen Sie die folgenden digitalen gesundheitsbezogenen Informationsangebote?)

Information services:

- Websites on the topic of health
- Social media (online forums, Facebook, Instagram)
- Digital devices related to health or medical care (e.g., fitness trackers, smartwatches)
- Health apps on mobile phones (e.g., food diary)
- Digital interactions with healthcare providers, e.g., submitting invoices for treatments via email
- Digital services to support home care (e.g., fall prevention, therapy planning)
- Telemedicine services, e.g., telemedicine consultations/online video consultations
- Appointment scheduling via an online portal

Answer categories:

0 = never

1 = rarer

2 = weekly

3 = daily
